# Upregulated Krüppel-like factor 5 promotes hepatocellular carcinoma progression by activating Wnt3a signaling

**DOI:** 10.1016/j.gendis.2025.101685

**Published:** 2025-05-15

**Authors:** Wenli Sai, Jie Yang, Liwei Qiu, Min Xu, Min Yao, Dengfu Yao

**Affiliations:** a Research Center of Clinical Medicine, Affiliated Hospital of Nantong University & Department of Immunology, Medical School of Nantong University, Nantong, Jiangsu 226001, China; b Department of Biology, Life Secince School of Nantong University, Nantong, Jiangsu 226019, China

Early diagnosis and effective treatment of hepatocellular carcinoma (HCC) are crucial. Hepatocarcinogenesis involves multiple genes and processes with complicated mechanisms. Recently, zinc finger proteins (ZNFs) have been found to constitute the largest family in the human genome and are functional proteins involved in regulating cell differentiation, embryonic development, and a variety of diseases. Additionally, the regulation of target gene transcription factors can vary with environmental stimuli and cell type. Complex ZNFs with up to 13 Krüppel-like transcription factor (KLF) abnormalities are related to the progression of multiple types of tumors. Here, we report a new molecular marker, KLF5. The up-regulated KLF5 mRNAs in human HCC tissues were confirmed via The Cancer Genome Atlas (TCGA) database, and abnormally increased KLF5 was analyzed in human HCC tissues via multicolor immunofluorescence technology. Additionally, the clinicopathological features of patients with KLF5 overexpression were TNM stage, tumor size, alpha fetoprotein (AFP) level, portal vein thrombosis, and HBV infection. High KLF5 expression was negatively correlated with the prognosis of HCC patients. Clinically, increased circulating KLF5 levels might be helpful in HCC diagnosis or differential diagnosis from patients with benign and malignant liver diseases. Mechanistically, KLF5 could be co-expressed with Wnt3a in the same HCC cells and might promote HCC progression via cross-talk.

As a transcriptional activator, KLF5 can regulate gene transcription, the cell cycle, and cell proliferation and differentiation and plays a pivotal role in the regulation of cancer stem-like cells and the promotion of cell growth and metastasis by activating the phosphoinositide 3-kinase (PI3K)/protein kinase B (AKT)/Snail signaling pathway in HCC. Aberrant KLF5 expression is associated with the malignant progression of liver diseases through direct or indirect effects at the transcriptional or posttranslational level.^1^ Abnormal KLF5 levels have been reported in benign and malignant liver diseases. However, the role of oncogenic KLF5 as a novel biomarker and its molecular function in HCC progression remain to be identified. In this study, KLF5 expression in HCC tissues and serum from patients with chronic liver diseases was further investigated to analyze the related clinicopathological characteristics and to determine the value of KLF5 for HCC diagnosis and prognosis (Fig. 1). Moreover, the biological functions of KLF5 and associated signaling pathways were verified via data from a bioinformatics database.

**Figure 1:**
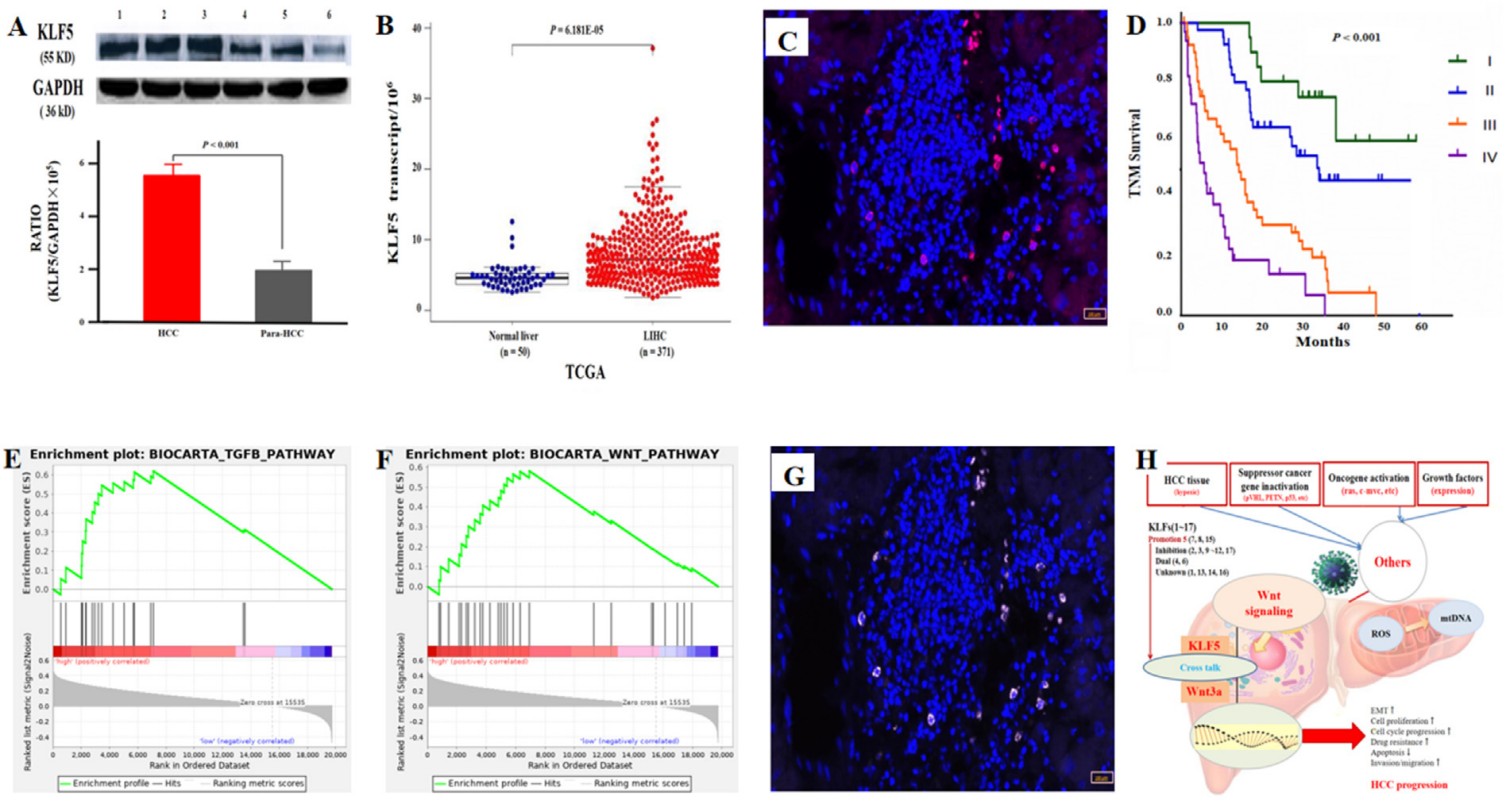
Oncogenic KLF5 promoted HCC progression by activating Wnt3a signaling. **(A)** Comparative analysis of KLF5 expression by western blotting (top) and the ratios of hepatic KLF5 to glyceraldehyde-3-phosphate dehydrogenase (GAPDH) (bottom) between HCC (*n* = 40) and para-HCC (*n* = 40) tissues. **(B)** Comparative analysis of KLF5 mRNA levels between liver hepatocellular carcinoma (LIHC) (*n* = 371) and normal liver tissues (*n* = 50) from the TCGA database (https://www.cancer.gov). **(C)** KLF5 in human HCC tissues was detected via multicolor immunofluorescence technology. **(D)** KLF5 expression in HCC was associated with TNM stage and the 5-year survival rate of HCC patients. **(E)** KLF5 mRNA expression was correlated with TGF-β signaling according to Biocarta analysis (https://cn.bing.com/dict/biocarta). **(F)** KLF5 mRNA expression was correlated with Wnt signaling according to Biocarta analysis. **(G)** Clinical validation of Wnt3a plus KLF5 co-expression in HCC tissues via multicolor immunofluorescence technology. **(H)** Hepatic KLF5 and Wnt3a were clearly expressed in the same cells, and their cellular colocalization might represent another novel mechanism underlying KLF5 promoting HCC progression. KLF5, Krüppel-like transcription factor 5; HCC, hepatocellular carcinoma.

Hepatocarcinogenesis involves multiple genes and processes with complicated mechanisms. Recently, KLFs (17 members in total), which constitute the largest ZNF family in the human genome, were shown to be involved in regulating cell differentiation, embryonic development, and disease-related functional proteins in different cancers.^2^ KLFs are divided into the following groups according to their biological functions: promoting (KLF5; Fig. 1A), inhibiting, dual, and unknown functions. To understand the relationship between up-regulated KLF5 and HCC (Fig. 1B), the intracellular localization of KLF5 in human HCC tissues (Fig. 1C) was determined, and the circulating levels of KLF5 in patients with chronic liver diseases were quantitatively investigated to analyze its clinical value and explore the mechanism underlying KLF5 promoting HCC progression.

Aberrant KLF5 expression is involved in tumor biological activity, induces pluripotent stem cells, and maintains an embryonic stem cell state. KLF5 contains a zinc finger domain that binds to target DNA and regulates not only physiological processes, such as cell proliferation, development, differentiation, and embryonic development, but also the progression of multiple diseases and conditions, including HCC and inflammation.^3^ In this study, significantly high KLF5 expression in the HCC group was associated with TNM stage, tumor size, AFP level, portal vein thrombosis, HBV infection, and the 5-year survival rate of HCC patients (Fig. 1D). These findings suggest that high KLF5 expression not only promotes HCC progression but also might be a prognostic marker for HCC patients.

Abnormal KLF5, a soluble protein similar to AFP in tissues, can be directly secreted into the circulation.^4^ In this study, serum KLF5 levels were quantified in patients with chronic liver disease, and significantly high KLF5 levels were detected in HCC patients. However, the levels of both KLF5 and AFP in the serum of HCC patients were markedly greater than those in the serum of patients with chronic hepatitis or liver cirrhosis and normal controls. However, the use of KLF5 as a new tumor marker was superior to AFP in terms of specificity and sensitivity, with complementary diagnostic value for HCC, especially in patients with low AFP levels. These data indicate that KLF5 might be a novel biomarker for the diagnosis or differential diagnosis of HCC and for distinguishing benign and malignant liver diseases. KLF5 is negatively correlated with noncoding RNAs that can regulate key transcription factors in liver cancer stem cells.^5^ KLF5 acetylation plays the opposite role in HCC growth, as deacetylated KLF5 exhibits protumor activity, and blocking transforming growth factor beta (TGF-β) signaling (Fig. 1E) attenuates the inhibitory activity of KLF5. In this study, hepatic KLF5 and Wnt3a (Fig. 1F) were clearly expressed in the same cells, and because of their cellular localization, cross-talk between KLF5 and Wnt3a (Fig. 1G) might represent another novel mechanism (Fig. 1H) closely related to HCC progression.

According to the reported literature,^2^ the roles of KLFs in HCC can be divided into four categories: promoting (KLF5, KLF7, KLF8, and KLF15), inhibiting (KLF2, KLF3, KLF9 ∼ KLF12, and KLF17), dual (KLF4 and KLF6), and unknown (KLF1, KLF13, KLF14, and KLF16) functions. KLFs are a group of conserved zinc finger-containing transcription factors that are involved in HCC development, including cell proliferation, differentiation, and apoptosis. It is speculated that oncogenic KLF5, together with the oncogene Wnt3a, should accelerate the malignant transformation of hepatocytes or the progression of HCC. However, further basic and clinical studies are needed to determine whether KLF5 could serve as a novel diagnostic or prognostic biomarker or as a therapeutic target for HCC.

## Open-access

This article is an open-access article that was selected by an in-house editor and fully peer-reviewed by external reviewers. It is distributed in accordance with the Creative Commons Attribution Non Commercial (CCBY-NC4.0) license, which permits others to distribute, remix, adapt, build upon the work noncommercially, and license their derivative works on different terms, provided the original work is properly cited and the use is noncommercial.

See: http://creativecommons.org/licenses/bync/4.0/

## CRediT authorship contribution statement

**Wenli Sai:** Methodology, Investigation, Data curation, Conceptualization. **Jie Yang:** Methodology, Investigation, Conceptualization. **Liwei Qiu:** Methodology, Investigation, Conceptualization. **Min Xu:** Methodology, Investigation, Formal analysis. **Min Yao:** Writing – review & editing, Validation, Methodology, Funding acquisition. **Dengfu Yao:** Writing – review & editing, Writing – original draft, Funding acquisition, Conceptualization.

## Ethics declaration

The study protocol was approved (TDFY2018-025) by the Medical Ethics Committee of the Affiliated Hospital of Nantong University, China, and prior written informed consent was obtained from HCC patients according to the Helsinki Declaration of the World Medical Association.

## Funding

This study was supported by the 10.13039/501100001809National Natural Science Foundation of China (No. 81673241, 31872738).

## Conflict of interests

The authors declared no conflict of interests.
