# Supplementary material for: Upregulated Krüppel-like factor 5 promotes hepatocellular carcinoma progression by activating Wnt3a signaling
Source: Genes Dis. 2025 May 15;12(6):101685. doi: 10.1016/j.gendis.2025.101685 (PMC12281284; doi:10.1016/j.gendis.2025.101685)
Supplement: Multimedia component 1 [file mmc1.docx]

**Appendix:**

**Materials & methods**

***Bioinformatics analysis*** KLF5 mRNAs in HCC or adjacent normal tissues were abstracted from the Cancer Genome Atlas (TCGA, [https://www.cancer.gov](https://www.cancer.gov/about-nci/organization/ccg/)), online data that provided crucial interactive and customizable functions, such as differential levels of KLF5 transcription in HCC and adjacent tissues, patient survival and correlation. Based on enrichment analysis of the Kyoto Encyclopedia of Genes and Genomes (KEGG, [https://www.genome.ad.jp/kegg)](https://www.KEGG.jp),) and Biocarta (http://www. biocarta.com), major signal pathways are involved in KLF5 promoting cancer progression.

***Liver tissues and clinical data*** This study was approved by the Ethics Committee permission (TDFY2018-025) at the Affiliated Hospital of Nantong University, China from Jan 2014 to Dec 2018, and the prior written informed consent was obtained from HCC patients according to the Helsinki Declaration of World Medical Association. Total 85 pairs of self-controlled HCC and their para-cancerous tissues (2 cm to cancer) were collected from patients with HCC after post-operation, frozen in liquid nitrogen, and kept at -85 ℃ until used. According to their medical records, the cases were 64 males and 21 females with 25 ~ 67-yr-old (average 48.1 ± 12.9 yrs). There were 75 cases with positive HBV surface antigen (HBsAg), 55 cases with tumor size ≥ 3.0 cm, 60 cases with AFP ≥ 20 ng/mL and 42 cases with vascular invasion. According to the 2019 edition of the diagnosis and treatment of primary liver cancer, there were well (I), medium (Ⅱ), and poor (Ⅲ) differentiation. Stagings were 43 cases at I ~ II and 42 at III ~ IV on the tumor- node-metastasis (TNM) classification of the International Union Against Cancer. All patients underwent histopathology and did not receive radiation or chemotherapy prior to surgery. HCC patients had regular follow-up from operation to death until Dec 2018. Diagnosis of HCC was confirmed with the criteria set by the Chinese National Collaborative Cancer Research Group.

***Blood*** Blood were collected from hospitalized patients with chronic liver diseases including HCC (n = 85), chronic hepatitis (CH, n = 40) and liver cirrhosis (LC, n = 40), with healthy persons as normal control (NC, n = 40) group. After serum were separated and stored in -80 ℃ for use. All patients had complete follow-up data and were diagnosed by pathology, serum AFP and imaging. The liver and kidney function, blood glucose and blood lipid of the NC group, AFP concentration were less than 20 ng/mL as a normal reference range.

***Tissue microarrays (TMA)*** TMA were made by the Departments of Pathology, Affiliated Hospital of Nantong University, China, containing formalin-fixed and paraffin-embedded specimens from 85 HCC tissues and their para-cancerous tissues. Tissue cores (1.5 mm) from representative areas were constructed into slices.

***Multiplex immunofluorescence (MIF) staining*** MIF staining was performed with the OPAL IHC kit from PerkinElmer (ABSIN, Shanghai, CN). Slides following steps of multiplex IHC were followed consecutively for each marker: blocking was performed with antibody diluent, followed by incubation with 1^st^ antibody for 1 h, detected using OPAL Polymer HRP antibodies (Waltham, MA, USA), and visualized using OPAL tyramide signal amplification plus agent, after which the section was placed in EDTA buffer (pH8.0) and heated using microwave. Antibodies against KLF5 (ABCAM, UK, 1:1,000), and Wnt3a (ABCAM, UK, 1:1000) were used as primary antibodies. All antibodies were used in order and marked with a specific color. In the first set of assays, KLF5 and Wnt3a were identified, with anti-KLF5 marked with red (Cy5, 650) and anti-Wnt3a marked with white (FITC, 570). After staining, the specimens were washed and then sealed with glycerine. All of the above procedures were conducted at 25 ℃, and slides were maintained in the dark. Slides in which primary antibodies was omitted were used as negative controls. KLF5 expression were calculated with the Image-Pro Plus 6.0 software (Media Cybernetics, Rockville, MD, USA). Blinded evaluations of KLF5 staining and independent observation by two independent pathologists were carried out simultaneously, and scores were calculated by staining intensities and positive cell number.

***Analysis of MIF staining*** Hepatic KLF5 expressions were divided into low or high levels, and KLF5 intensities were divided into 4 categories: negative (-, 0 sore); weakly positive (+, 1 sore); moderate (++, 2 sores); and strongly positive (+++, 3 sores), respectively. Images were acquired under a light microscope with a 40 × objective lens (Olympus, Japan). KLF5 expressions in TMA sections were used by the Vectra 3.0 automatic quantitative pathology imaging system. Finally, the INFORM software was used to score the staining. TMA slices were observed and photographed under microscope and analyzed by ImagePro Plus v6.0 software with integral optic density (IOD) value.

***Western blotting*** Total protein was extracted from livers and quantified by the BCA (Biyuntian Biotech. Co., Ltd., CN) kit. Separated and concentrated gel were prepared and fixed in electrophoresis tank. Pre-stained samples and markers were added to the tank. After the markers were added into separation gel, voltage was adjusted to 120 V to continue electrophoresis until end. Polyvinylidene fluoride (PVDF) membrane was transferred, added 5 % skim milk in TBST solution, and slowly sealed at 25 ℃ for 2 h, covered with KLF5 (ABCAM, UK, 1:1,000) or glyceraldehyde-3-phosphate dehydro- genase (GAPDH) antibodies solution (ABCAM, UK) overnight at 4 ℃, and covered with the 2^nd^ antibodies solution for 2 h at 25 ℃. Added solution A with B were mixed at 1:1, dropped evenly on film (avoiding light) for 2 min.

***Enzyme linked immunosorbent assay (ELISA)*** Serum KLF5 (ng/mL) or liver KLF5 specific concentration (ng/mg wet tissue) were quantitatively detected according to the manual of human KLF5 ELISA kit (Qiming Biotech. Co., Shanghai, CN). Averaged absorbance (A) at 450 nm (n = 3) was measured for standard, control and sample. KLF5 levels were calculated based on the corresponding standard curve.

***Statistical analysis*** Data were expressed with the mean ± standard deviation (M ± SD). GraphPad Prism 7.0 was used to conduct statistical analysis. Multiple comparisons were analyzed by ANOVA followed by the *q* test. Data from pathological grades were analyzed using the *t* test. Positive ratios between groups were compared by the χ^2^ test. A *P* < 0.05 value was defined as statistically significant.
